# The Effects of Smartphone Addiction on Academic Performance Among Undergraduate Medical Students in Karnataka, India: A Multi-centric Study

**DOI:** 10.7759/cureus.62796

**Published:** 2024-06-20

**Authors:** Qudsia Zeerak, Mohammed Imran, Kahkashan Azeez, Tejaswi H Lokanathan, Imaad Mohammed Ismail

**Affiliations:** 1 Community Medicine, Yenepoya Medical College, Mangaluru, IND; 2 Community Medicine, SS Institute of Medical Sciences and Research Centre, Davanagere, IND; 3 Physiology, Yenepoya Medical College, Mangaluru, IND; 4 Anatomy, Adichunchanagiri Institute of Medical Sciences, Mandya, IND

**Keywords:** medical students, risk factors, academic performance, mobile phone addiction, smartphone addiction

## Abstract

Background and objective

While smartphones offer various benefits, addiction to them among young people poses a serious problem for parents and educators globally. Several studies have tried to assess the impact of the excessive use of/addiction to smartphones on students' overall academic performance. While a few studies have found a positive impact, most have observed a negative impact, mainly in the form of deterioration of mental and physical health and academic performance of students. In light of this, we conducted this study in three medical colleges in Karnataka, India with the objectives of determining the effect of smartphone addiction on the academic performance of undergraduate medical students and to identify the risk factors associated with it.

Material and methods

This cross-sectional study, conducted between July 2022 and October 2023, involved undergraduate medical students from Yenepoya Medical College, Subbaiah Institute of Medical Sciences, and Adichunchanagiri Institute of Medical Science. A total of 481 students were enrolled by stratified random sampling. Data were collected with the help of Google Forms by using a pre-tested questionnaire. Mobile phone addiction was assessed by using the smartphone addiction scale-short version (SAS-SV).

Results

Among 481 students, 211 (43.9%) were found to be mobile phone addicts. An independent t-test showed that academic performance was negatively affected in students who were mobile phone addicts. Multivariate logistic regression analysis revealed several factors significantly associated with mobile phone addiction, including male gender, regular mobile gaming, seeking recognition/popularity through social media, frequent smartphone checking, perceiving smartphone use as more attractive than other activities, limited co-curricular activities, and increased smartphone use during periods of depression or anxiety.

Conclusions

Based on our findings, smartphone addiction has a negative impact on the academic performance of students. To mitigate this issue, educational institutions should integrate efforts to tackle smartphone addiction into their curricula and prioritize addressing the identified risk factors.

## Introduction

We are living amidst a technological revolution, wherein electronic devices, particularly smartphones, are among the most popular and handy gadgets used in daily life. Since the release of the first publicly available smartphone in 2007, this industry has grown exponentially in terms of technological advancement and ease of availability [1]. Consequently, smartphones have become user-friendly, inexpensive, and highly evolved, paving the way for their global usage. Moreover, easy internet access on this device has ushered in an era of internet revolution. There are an estimated 4.9 billion smartphone users worldwide in 2024, which is expected to reach 6.4 billion by 2029 [2].

As a result of this smartphone revolution, a variety of activities like the use of social media applications, web surfing for educational and recreational purposes, gaming, electronic communication, instant messaging, streaming content, and online transactions can be easily performed at one’s convenience anywhere in the world using smartphones. Smartphones and the internet have revolutionized the field of education too. Thanks to easy internet access, students can find every type of study material, use various e-learning applications, take online exams, and expand their academic skills in innumerable other ways. However, in recent years, indiscriminate and compulsive use of mobile phones has been observed in all age groups, especially among school and college students, termed “smartphone addiction”. Other terms like “smartphone overuse”, “mobile phone addiction” and “problematic mobile phone use” have also been used to describe this phenomenon. It is defined as an impulse control disorder manifesting as a strong urge to use a smartphone despite knowing its negative effects (i.e., neglecting other aspects of life due to the constant urge and excessive use) [3].

Despite the benefits of smartphones in education, smartphone addiction and its associated internet addiction pose significant challenges to parents, guardians, and educators worldwide. Many students are seen spending long hours on their phones, chatting with friends, using social media applications, browsing the internet, and watching entertainment-related content, in addition to using them for studies. Numerous studies have tried to assess the impact of this usage on students' overall academic performance. While some studies have reported smartphones having a positive effect on students' academic performance, most have found a negative impact [4-7]. It has been observed that the indiscriminate and excessive use of mobile phones has adversely affected psycho-social behavioral traits and mental health, interpersonal relationships, physical health, and academic performance among students in recent years [8].

Today's medical students will become the doctors of tomorrow. These students are particularly susceptible to smartphone addiction, often possessing high-quality smartphones with fast internet connections. Additionally, they may reside away from parental supervision and face significant academic pressure. It is crucial to understand the impact of smartphone addiction on the academic performance of medical students and to identify the responsible factors, as this forms the basis for implementing measures to mitigate it. However, there is a general dearth of research on this topic, both globally and in India. Hence, this study was undertaken in three medical colleges in Karnataka with the objectives of determining the effect of smartphone addiction on the academic performance of undergraduate medical students and identifying the risk factors associated with it.

## Materials and methods

This multi-center cross-sectional study was conducted from July 2022 to October 2023 and involved undergraduate students from three medical colleges in Karnataka State: Yenepoya Medical College (Mangalore District), Subbaiah Institute of Medical Sciences (Shivamogga District), and Adichunchungiri Institute of Medical Science (Mandya District). These institutions are situated in diverse geographic areas of the State and are affiliated with different universities. Ethical approval was obtained from the institutional ethics committees, and permission to conduct the study was granted by the principals of the institutes.

The sample size was calculated by using the formula, n=z^2^pq/l^2^ where z is the z-score corresponding to the desired confidence level, p is the estimated prevalence, q is 1-p, and l is the desired margin of error. We adopted a 99% confidence interval, a prevalence of 87%, and a relative error of 5%. After determining a target sample size of 397, an additional 30% was added to account for potential non-response, resulting in a sample size of 516, with 172 students each from the three colleges. Thus a minimum sample size of 397 was required for the study. Stratified random sampling was employed at the college level, including undergraduate medical students from the first year to the final year, while interns were excluded.

Data were collected with the help of Google Forms by using a pre-tested, semi-structured questionnaire validated by three subject experts. The Google Form link was sent to the selected students via email or WhatsApp. The students were first asked to read the participant information sheet and, if they consented, they could proceed to answer the questions. The questionnaire collected information on the sociodemographic data of the student, smartphone usage using the smartphone addiction scale-short version (SAS-SV), academic performance, and risk factors associated with smartphone addiction. The SAS-SV developed by Kwon et al. consists of 10 questions all weighted equally on a 6-point scale, with cutoff values of 31 for boys and 33 for girls to determine smartphone addiction [9]. The overall percentage of marks in recent university exams was considered for academic performance evaluation, and, for first-year students, the recent internal exam results were considered.

The data analysis involved downloading the Google Form responses as a CSV file, followed by refining, coding, and subsequent analysis using IBM SPSS Statistics version 26 (IBM Corp., Armonk, NY). Descriptive statistics like mean, frequency, and proportions were applied and statistical tests such as independent t-test and logistic regression were used. Logistic regression analysis was done to identify the risk factors associated with smartphone addiction. The variables with a p-value less than 0.25 in univariate analysis were considered potential independent risk factors and were further analyzed together in multivariate regression analysis. Variables in multivariate analysis with p-values less than 0.05 were considered to have a significant effect on smartphone addiction.

## Results

The study included 481 students who completed the questionnaire across the three medical colleges (Table 1). Among them, 302 (62.8%) were females while the rest were males. The mean (±SD) age of the participants was 20.9 (±1.5) years. The majority of participants (84%) resided in hostels, while the rest were day scholars who resided with family, alone, or with friends (Table 2).

**Table 1:** Distribution of students from the selected medical colleges (N=481)

Students	Yenepoya Medical College	Subbaiah Institute of Medical Sciences	Adichunchungiri Institute of Medical Sciences
First year	42	41	41
Second year	41	39	39
Third year part 1	42	41	40
Third year part 2	38	37	40
Total	163	158	160

**Table 2:** Gender and residence-wise distribution of the study population (N=481)

Variable		Frequency	%
Gender	Male	179	37.2
	Female	302	62.8
Place of stay	Hostel	404	84.0
	Day scholar, staying with family	43	8.9
	Day scholar, staying alone	15	3.1
	Day scholar, staying with friends	19	4.0

Of the 481 students, 211 (43.9%) were found to be addicted to smartphones. The mean (±SD) academic scores in the smartphone addiction group and the non-addict group were 64.6 (±9.4) and 66.1 (±8.3) respectively. Since the scores were from students across different universities having different question papers, standardization was performed by subtracting the scores from the mean score of their college and dividing it by the SD values of their college (Table 3). An independent t-test was applied, which showed that academic performance was negatively affected in students who were smartphone addicts.

**Table 3:** Overall and standardized academic scores of smartphone addicts and non-addicts (N=481) ^*^Independent t-test p-value of 0.044 SD: standard deviation

Study variable		N	Mean	SD
Overall academic scores	Addicts	211	64.6	9.38
	Non-addicts	270	66.1	8.26
Standardized academic scores^*^	Addicts	211	-0.097	1.055
	Non-addicts	270	0.089	0.952

Univariate logistic regression analysis was performed to check for predictors of smartphone addiction (Tables 4-5). On applying multivariate logistic regression, it was found that the male gender, regular engagement with mobile phone games, use of social media to gain recognition/popularity, frequent smartphone checking, finding the smartphone more attractive than other things, limited availability of co-curricular activities, and increased mobile phone usage during periods of depression or anxiety were significantly associated with smartphone addiction.

**Table 4:** Logistic regression analysis of demographic characteristics and smartphone usage as risk factors for smartphone addiction (N=481) CI: confidence interval; OR: odds ratio

Variable	Total	Smartphone addicts	Univariate analysis		Multivariate analysis	
	N	N (%)	Unadjusted OR (95% CI)	P-value	Adjusted OR (95% CI)	P-value
Age in years			1.08 (0.95-1.21)	0.239	1.02 (0.88-1.19)	0.765
Gender						
Male	179	97 (54.2)	1.95 (1.34-2.88)	<0.001	2.42 (1.47-3.99)	<0.001
Female	302	114 (37.7)	Reference		Reference	
Access to smartphone with free/low-cost and high-speed internet						
Most of the time	306	136 (44.4)	1.33 (0.74-2.40)	0.336	-	-
Restricted duration	119	54 (45.4)	1.39 (0.72-2.66)	0.327	-	-
No access	56	21 (37.5)	Reference		-	-
Mobile phone gaming						
Regular	52	30 (57.7)	1.91 (1.04-3.51)	0.037	0.43 (0.18-1.01)	0.05
Occasional	196	84 (42.9)	1.05 (0.72-1.55)	0.798	0.85 (0.51-1.39)	0.51
Do not game on mobile	233	97 (41.6)	Reference		Reference	
Online shopping using a smartphone						
Regular	74	45 (60.8)	1.97 (1.06-3.66)	0.033	1.36 (0.60-3.07)	0.456
Occasional	314	125 (39.8)	0.84 (0.53-1.34)	0.461	0.69 (0.38-1.26)	0.229
Do not shop online	93	41 (44.1)	Reference		Reference	
Use of social media on a smartphone						
Very frequently	299	158 (52.8%)	2.08 (0.80-5.36)	0.129	0.77 (0.24-2.45)	0.662
Sometimes	162	46 (28.5%)	0.74 (0.28-1.96)	0.736	0.53 (0.16-1.70)	0.285
Do not use	20	7 (35.0%)	Reference		Reference	
Use of social media to get importance						
To a great extent	35	28 (80.0%)	7.40 (3.12-17.53)	<0.001	4.90 (1.70-14.09)	0.003
To some extent	147	78 (53.1%)	2.09 (1.48-3.12)	<0.001	1.33 (0.80-2.21)	0.268
Do not use	299	105 (35.1%)	Reference		Reference	
Use of smartphone for entertainment (movies, web series, etc.)						
Frequent	255	132 (51.8%)	2.68 (1.14-6.31)	0.024	1.00 (0.33-2.96)	0.994
Sometimes	198	71 (35.9%)	1.40 (0.59-3.33)	0.451	0.95 (0.32-2.77)	0.918
Do not use	28	8 (28.6%)	Reference		Reference	
The habit of frequently checking smartphone						
Yes	286	168 (58.7%)	5.03 (3.33-7.60)	<0.001	2.46 (1.47-4.11)	<0.001
No	195	43 (22.1%)	Reference		Reference	
Use of smartphone in bed before sleeping						
Regular	296	161 (54.4%)	3.22 (1.50-6.90)	0.003	1.23 (0.46-3.1)	0.670
Occasional	148	40 (27.0%)	1.0 (0.44-2.25)	1	0.61 (0.22-1.66)	0.334
Do not use	37	10 (27.0%)	Reference		Reference	
Average duration of smartphone use per day in hours			1.27 (1.16-1.39)	<0.001	1.08 (0.96-1.21)	0.206

**Table 5:** Logistic regression analysis of social and psychological variables as risk factors for smartphone addiction (N=481) CI: confidence interval; OR: odds ratio

Variable	Total	Smartphone addicts	Univariate analysis		Multivariate analysis	
	N	N (%)	Unadjusted OR (95% CI)	P-value	Adjusted OR (95% CI)	P-value
Smartphones more attractive as compared to other things in life (chatting with friends or time with family or hobby)						
Yes	88	74 (84.1%)	9.87 (5.38-18.13)	<0.001	4.82 (2.34-9.91)	<0.001
No	393	137 (34.9%)	Reference		Reference	
Less availability of family members or friends to talk						
Yes	140	89 (63.6%)	3.13 (2.08-4.71)	<0.001	1.45 (0.84-2.52)	0.184
No	341	122 (35.8%)	Reference		Reference	
Availability of co-curricular activities (sports, book clubs, music, crafts, etc.) at your institution or nearby area						
Many activities are available	98	41 (41.8%)	1.23 (0.72-2.08)	0.440	1.04 (0.52-2.01)	0.900
Few activities available	242	118 (48.8%)	1.62 (1.06-2.50)	0.024	1.80 (1.05-3.08)	0.033
Not available	141	52 (36.9%)	Reference		Reference	
Regular use of smartphones as there is nothing else interesting to do in your life						
Yes	240	127 (52.9%)	2.10 (1.45-3.03)	<0.001	0.98 (0.59-1.63)	0.931
No	241	84 (34.9%)	Reference		Reference	
Tendency to use smartphone more when feeling depressed or anxious						
Yes	288	153 (53.1%)	2.64 (1.80-3.88)	<0.001	1.79 (1.05-3.06)	0.033
No	193	58 (30.1%)	Reference		Reference	
Use of mobile phone as an escape from academic stress						
Yes	297	154 (51.9%)	2.40 (1.63-3.53)	<0.001	0.69 (0.39-1.23)	0.211
No	184	57 (31.0%)	Reference		Reference	
Influenced to frequently use smartphones as people around are always using it						
Yes	238	132 (55.5%)	2.59 (1.78-3.74)	<0.001	1.56 (0.98-2.48)	0.062
No	243	79 (32.5%)	Reference		Reference	
Use of mobile phone in public to avoid conversations						
Frequently	149	94 (63.1.%)	4.77 (2.69-8.46)	<0.001	1.88 (0.90-3.95)	0.093
Sometimes	241	93 (38.6%)	1.75 (1.03-3.00)	0.039	1.11 (0.58-2.12)	0.744
Do not use	91	24 (26.4%)	Reference		Reference	

## Discussion

Smartphone use by medical students has its pros and cons. This multi-centric study was undertaken to assess the effect of smartphone addiction on academic performance and identify the factors associated with smartphone addiction among undergraduate medical students of Karnataka, India. The study involved 481 students and found that 43.9% were addicted to smartphones and the addiction had a negative effect on their academic performance. Factors such as male gender, regular mobile gaming, seeking importance through social media, frequent smartphone checking, perceiving smartphones as more attractive than other activities, limited co-curricular activities, and increased smartphone use during periods of depression or anxiety were associated with smartphone addiction.

The study invited 516 medical students, of which 481 participated, indicating a healthy 93.2% response rate. A multi-center design was selected to comprehensively represent students from three distinct universities. This approach enhances the reliability of the study and allows for the findings to be effectively utilized in policy advocacy efforts. About two-thirds of the study participants were females, reflecting the general trend in Karnataka State where the proportion of females enrolled for medical courses is higher than males. Most of the students were aged 18-23 years and about 90% of the students were staying in hostels, alone, or with friends outside the hostel. This is common among medical students as they often relocate to a different District or State for their medical education.

Smartphone addiction was observed in 43.9% of medical students. Other studies involving Indian medical students have reported this in the range of 34.8%-54.2% [10-14]. It must be noted that this difference is partly due to the different scales used to asses smartphone addiction in studies. Nevertheless, our findings are in light with those of other studies highlighting that smartphone addiction is highly concerning. The study observed smartphone addiction detrimentally affected the academic performance of students. Unfortunately, no similar studies among Indian medical students were available to compare and contrast the findings. A study by Tian et al. among medical students in China observed that mobile addiction among medical students resulted in decreased learning dedication and learning performance [7]. Similar findings were also reported in a study by Alharbi et al. in Saudi Arabia [15]. Studies conducted at non-medical institutions have also indicated that smartphone addiction is associated with diminished academic performance [5,6,8,16].

Smartphone addiction affects students' learning both in the classroom and outside. A study by Khatgaonkar et al. from Pune, India reported that 38% of nursing students used mobile phones while a lecture was going on in class [17]. Texting, listening to music, accessing social media, or even gaming during lectures is not uncommon in medical students. When studying at the hostel or at home, mobile-addicted students often check their mobile phones for notifications or use apps of their interest, which breaks their concentration, reducing their cognitive performance as well as their overall reading time [18]. Thus, both the quality and quantity of reading are affected. The impact of smartphone addiction on academic performance is particularly significant within the medical student community, given their role as future healthcare professionals responsible for patient care and well-being, making it imperative to address this issue.

Given that smartphone addiction leads to decreased academic performance, it is important to know why the students are getting addicted, and what are the risk factors associated with mobile addiction. This study observed that males (54.2%) had significantly higher addiction to smartphones compared to females (37.7%). A global literature search on this topic revealed mixed results with some studies finding males at higher risk of mobile addiction whereas the others found females to be at higher risk [7,13,19,20]. Thus, we can infer that we need to educate and protect all genders from this hazard of mobile addiction. An interesting finding was revealed in a study by Nayak et al., where they observed that smartphone addiction was more prevalent in females; however, the negative effect on academic performance was more profound among male students [16].

Regular mobile phone gaming was the next factor associated with smartphone addiction. Many other studies have reported similar findings, especially those from South Korea where mobile gaming is consistently associated with mobile addiction [21-23]. Mobile phone gaming can lead to addiction due to several factors such as escape from reality, allowing players to immerse themselves in another universe, stimulating brain reward pathways triggering dopamine release and making players want to keep playing, desire to reach the next level/achievement in the game, and the immersive nature of modern gaming with attractive visuals and audio [24]. Additionally, its accessibility and user-friendly interface contribute to habitual use.

Social media platforms enable people to connect and network with people online and allow them to share content and express opinions. However, excessive use of social media, especially to get noticed by others, was found to be a risk factor for smartphone addiction in the present study. This problem has also been reported in studies conducted in other parts of the world [22,23,25]. The present study also identified frequent smartphone checking as another significant factor associated with addiction. Comparable findings were reported in other studies, including a meta-analysis conducted by Sunday et al [5,6,10,21]. Dr. Anna Lembke, an addiction expert, likens smartphones to modern-day hypodermic needles, where each social media like, swipe, and tweet releases quick bursts of dopamine, reinforcing addictive behavior [26]. Frequent smartphone checking, often driven by FOMO (Fear of Missing Out), is primarily motivated by the need to stay updated on social media, along with entertainment, music, gaming, and news [27,28]. Checking phones for more than 20-30 times per day has been associated with addiction [6,10]. Moreover, once individuals begin checking their phones, they often end up spending more time than intended.

Spending time with family and friends improves the mental and social health of a person. Today, smartphones have become the best buddies for most people and the present study noted that people who found their smartphone more attractive compared with those time with family and friends, or engaging in their hobbies were at a higher risk for smartphone addiction. These findings are in line with other studies where poor social support and loneliness were found to be associated with mobile addiction [11,27,28]. Co-curricular activities such as sports, book clubs, music, and crafts are known to prevent addictions in general, which applies to smartphone addiction as well [29]. The present study observed mixed results in this regard and the limited availability of co-curricular activities was associated with smartphone addiction. Given the demanding nature of medical education, students often find little or no time for co-curricular pursuits. Consequently, students are frequently found either engrossed in medical books or turning to smartphones as a means of relaxation.

The study observed an association between depression/anxiety and smartphone addiction, which is supported by other research indicating a close relationship between smartphone addiction and mental health issues such as depression and anxiety [28]. A systematic review by Elhai et al. involving 23 articles found that depression and anxiety are related to problematic mobile phone use, which is more pronounced in the case of depression [30]. Depression and anxiety are associated with imbalances in neurotransmitters such as serotonin, dopamine, and norepinephrine. Smartphone use releases dopamine and individuals with depression or anxiety may seek this dopamine release more frequently through mobile phone use, leading to addictive behaviour. Moreover, individuals may also resort to smartphones as an escape mechanism from stressful situations; however, the reasons behind stress continue to persist, pushing them into a perpetuating cycle of depression/anxiety and smartphone addiction.

Strengths of this study include its multi-center design with a diverse representation of students from different universities and the utilization of a validated addiction scale to assess addiction, thereby ensuring objective measurement. One limitation of the study was that academic scores and risk factors for smartphone addiction were self-reported. Another limitation was that the temporal association between depression/anxiety and smartphone addiction was not assessed.

## Conclusions

Based on our findings, a high proportion of undergraduate medical students suffer from smartphone addiction, significantly affecting their academic performance. Factors associated with smartphone addiction include male gender, regular mobile gaming, seeking recognition/popularity through social media, frequent smartphone checking, perceiving smartphones as more attractive than other activities, limited co-curricular activities, and increased smartphone use during periods of depression or anxiety. The detrimental effect of smartphone addiction on the academic performance of medical students is concerning, as it can affect the competency of future doctors, jeopardizing healthcare delivery. Preventing this addiction requires educating and empowering students to use their smartphones wisely and promoting co-curricular activities. Governing bodies, such as the National Medical Commission, and universities, should integrate educational programs on smartphone addiction into the curricula and prioritize addressing the identified risk factors.

## Appendices

Questionnaire

I. Sociodemographic Details

1. Age in years:

2. Gender:   

     (1) Male

     (2) Female

     (3) Transgender

     (4) Other (please specify)

3. Year of MBBS:

     (1) 1st year

     (2) 2nd year

     (3) 3rd year, part 1

     (4) 3rd year, part 2 (final year)

4. Place of stay:

     (1) Hostel

     (2) Day scholar, staying with family/relatives

     (3) Day scholar, staying alone

     (4) Day scholar, staying with a friend

II. Smartphone addiction scale - short version (SAS-SV)

**Table 6:** Smartphone addiction scale

Sl. no.	Items	Strongly disagree	Disagree	Weakly disagree	Weakly agree	Agree	Strongly agree
1.	Missing planned work due to smartphone use						
2.	Having a hard time concentrating in class, while doing assignments, or while working due to smartphone use						
3.	Feeling pain in the wrists or at the back of the neck while using a smartphone						
4.	Won’t be able to stand not having a smartphone						
5.	Feeling impatient and fretful when I am not holding my smartphone						
6.	Having my smartphone in my mind even when I am not using it						
7.	I will never give up using my smartphone even when my daily life is already greatly affected by it						
8.	Constantly checking my smartphone so as not to miss conversations between other people on Twitter or Facebook						
9.	Using my smartphone longer than I had intended						
10.	The people around me tell me that I use my smartphone too much						

III. Questions related to academic performance

1. What was the overall percentage you got in your recent university exam? (If you are a first-year student, then enter the overall percentage you got in your recent internal exam - anatomy, physiology, biochemistry, and community medicine theory and practical exam) ___________

2. Do you think your smartphone usage is affecting your academic performance?

     (1) Yes, to a great extent

     (2) Yes, to some extent

     (3) No

     (4) Not sure

3. Do you often use the smartphone for non-academic purposes during class hours?

     (1) Yes, to a great extent

     (2) Yes, to some extent

     (3) No

4. Do you think smartphone usage is affecting your ability to concentrate while reading/listening to the teacher?

     (1) Yes, to a great extent

     (2) Yes, to some extent

     (3) No

     (4) Not sure

IV. Questions related to risk factors for smartphone addiction

1. Do you have access to a smartphone with free/low-cost and high-speed internet?

     (1) Yes, most of the time

     (2) Yes, restricted duration

     (3) No

2. Do you play mobile phone games?

     (1) Yes, regularly

     (2) Yes, occasionally

     (3) No

3. Do you shop online using a smartphone?

     (1) Yes, regularly

     (2) Yes, occasionally

     (3) No

4. Do you use social media on your smartphone?

     (1) Yes, very frequently

     (2) Yes, sometimes

     (3) No

5. Do you use social media to get importance/noticed by others?

     (1) Yes, to a great extent

     (2) Yes, to some extent

     (3) No

6. Do you use your smartphone for entertainment purposes such as watching movies, web series, etc.)?

     (1) Yes, very frequently

     (2) Yes, sometimes

     (3) No

7. Do you have the habit of frequently checking your smartphone so that you do not miss anything?

     (1) Yes

     (2) No

8. Do you use the smartphone in bed before sleeping?

     (1) Yes, regularly

     (2) Yes, occasionally

     (3) No

9. What is your average duration of smartphone use per day? ________ hours

10. Is the smartphone more attractive to you when compared to other things in life such as chatting with friends or spending time with family or your hobby?

     (1) Yes

     (2) No

11. Do you feel you have less availability of family members or friends to talk?

     (1) Yes

     (2) No

12. Are there any co-curricular activities (such as sports, book clubs, music, dance, painting and crafts, etc.) available in your institution or nearby area that you can utilize?

     (1) Yes, many activities are available

     (2) Yes, only a few are available

     (3) No

13. Do you think that you are using your smartphone often as there is nothing else interesting to do in your life?

     (1) Yes

     (2) No

14. Do you tend to use your mobile phone more when you are depressed or anxious because of any reason?

     (1) Yes

     (2) No

15. Do you find using a mobile phone an easy escape from academic stress?

     (1) Yes

     (2) No

16. Do you think that people around you are always using smartphones, hence you are also influenced to frequently use smartphones?

     (1) Yes

     (2) No

17. Do you use your mobile phone when you are in public to avoid conversations?

     (1) Yes, frequently

     (2) Yes, sometimes

     (3) No

## References

[1] Kinsbruner E, Reock J (2024). Kinsbruner E, Reock J: The history of phones and web technology development. https://www.perfecto.io/blog/evolution-of-smartphones-web.

[2] Turner A (2024). Turner A: How many people have smartphones worldwide. https://www.bankmycell.com/blog/how-many-phones-are-in-the-world.

[3] Kim E, Koh E (2018). Avoidant attachment and smartphone addiction in college students: the mediating effects of anxiety and self-esteem. Comput Hum Behav.

[4] Wang JC, Hsieh CY, Kung SH (2023). The impact of smartphone use on learning effectiveness: a case study of primary school students. Educ Inf Technol (Dordr).

[5] Sunday OJ, Adescope OO, Maarhuis PL (2021). The effects of smartphone addiction on learning: a meta-analysis. Comput Hum Behav Rep.

[6] Kalal N, Vel NS, Angmo S (2023). Smartphone addiction and its impact on quality of sleep and academic performance among nursing students. Institutional based cross-sectional study in Western Rajasthan (India). Invest Educ Enferm.

[7] Tian J, Zhao JY, Xu JM (2021). Mobile phone addiction and academic procrastination negatively impact academic achievement among Chinese medical students. Front Psychol.

[8] Achangwa C, Ryu HS, Lee JK, Jang JD (2022). Adverse effects of smartphone addiction among university students in South Korea: a systematic review. Healthcare (Basel).

[9] Kwon M, Kim DJ, Cho H, Yang S (2013). The smartphone addiction scale: development and validation of a short version for adolescents. PLoS One.

[10] Phukan I, Chetia A, Mahanta B (2022). Smartphone addiction and associated risk factors amongst undergraduate medical students in a medical college of Assam: a cross-sectional study. Int J Community Med Public Health.

[11] Verma N, Khan H, Singh A, Saxena R (2023). Smartphone addiction in medical students: association with perceived stress, personality factors and loneliness. Indian J Public Health.

[12] Devi K, Harshini S, Mageswari R (2022). Study of smartphone addiction and its predictors among medical college students in Puducherry, South India. Univ J Public Health.

[13] Chatterjee S, Kar SK (2021). Smartphone addiction and quality of sleep among Indian medical students. Psychiatry.

[14] Jahagirdar V, Rama K, Soppari P, Kumar MV (2021). Mobile phones: vital addiction or lethal addiction? Mobile phone usage patterns and assessment of mobile addiction among undergraduate medical students in Telangana, India. J Addict.

[15] Alharbi RS, Mohamed BA, Alshammari TM (2022). Addiction of smartphones and its relationship to academic achievement of medical students in Saudi Arabia [Preprint]. medRxiv.

[16] Nayak JK (2018). Relationship among smartphone usage, addiction, academic performance and the moderating role of gender: a study of higher education students in India. Comput Educ.

[17] Khatgaonkar C, Sayyad W, Mali D, Ramesh S, Mistry M (2020). Smartphone usage and its addiction among undergraduate nursing students. Indian J Forensic Med Toxicol.

[18] Skowronek J, Seifert A, Lindberg S (2023). The mere presence of a smartphone reduces basal attentional performance. Sci Rep.

[19] Parasuraman S, Sam AT, Yee SW, Chuon BL, Ren LY (2017). Smartphone usage and increased risk of mobile phone addiction: A concurrent study. Int J Pharm Investig.

[20] Lee H, Kim JW, Choi TY (2017). Risk factors for smartphone addiction in Korean adolescents: smartphone use patterns. J Korean Med Sci.

[21] Liu CH, Lin SH, Pan YC, Lin YH (2016). Smartphone gaming and frequent use pattern associated with smartphone addiction. Medicine (Baltimore).

[22] Lee SJ, Lee C, Lee C (2016). Smartphone addiction and application usage in Korean adolescents: effects of mediation strategies. Soc Behav Pers.

[23] Jeong SH, Kim H, Yum JY, Hwang Y (2016). What type of content are smartphone users addicted to? SNS vs. games. Comput Hum Behav.

[24] (2024). A new addiction on the rise: mobile game addiction. https://www.addictions.com/blog/a-new-addiction-on-the-rise-mobile-game-addiction/.

[25] Alotaibi MS, Fox M, Coman R, Ratan ZA, Hosseinzadeh H (2022). Smartphone addiction prevalence and its association on academic performance, physical health, and mental well-being among university students in Umm Al-Qura University (UQU), Saudi Arabia. Int J Environ Res Public Health.

[26] Waters J (2024). Waters J: constant craving: how digital media turned us all into dopamine addicts. https://www.theguardian.com/global/2021/aug/22/how-digital-media-turned-us-all-into-dopamine-addicts-and-what-we-can-do-to-break-the-cycle.

[27] Alinejad V, Parizad N, Yarmohammadi M, Radfar M (2022). Loneliness and academic performance mediates the relationship between fear of missing out and smartphone addiction among Iranian university students. BMC Psychiatry.

[28] Liu C (2023). The unique role of smartphone addiction and related factors among university students: a model based on cross-sectional and cross-lagged network analyses. BMC Psychiatry.

[29] Azam M, Ali A, Mattiullah J, Perveen N (2020). Physical activity, sports participation, and smartphone addiction in adolescent students: a systematic review. J Evid Based Psychother.

[30] Elhai JD, Dvorak RD, Levine JC, Hall BJ (2017). Problematic smartphone use: a conceptual overview and systematic review of relations with anxiety and depression psychopathology. J Affect Disord.

